# Innovative CAR-T Cell Therapy for Solid Tumor; Current Duel between CAR-T Spear and Tumor Shield

**DOI:** 10.3390/cancers12082087

**Published:** 2020-07-28

**Authors:** Yuna Jo, Laraib Amir Ali, Ju A Shim, Byung Ha Lee, Changwan Hong

**Affiliations:** 1 Department of Anatomy, Pusan National University School of Medicine, Yangsan 50612, Korea; yoona30@naver.com (Y.J.); laraib.meghjani@gmail.com (L.A.A.); fatehyun@hanmail.net (J.A.S.); 2 NeoImmuneTech, Inc., 2400 Research Blvd., Suite 250, Rockville, MD 20850, USA; BLee@neoimmunetech.com

**Keywords:** CAR-T, solid tumor, immunotherapy, T cell responses, tumor microenvironment

## Abstract

Novel engineered T cells containing chimeric antigen receptors (CAR-T cells) that combine the benefits of antigen recognition and T cell response have been developed, and their effect in the anti-tumor immunotherapy of patients with relapsed/refractory leukemia has been dramatic. Thus, CAR-T cell immunotherapy is rapidly emerging as a new therapy. However, it has limitations that prevent consistency in therapeutic effects in solid tumors, which accounts for over 90% of all cancer patients. Here, we review the literature regarding various obstacles to CAR-T cell immunotherapy for solid tumors, including those that cause CAR-T cell dysfunction in the immunosuppressive tumor microenvironment, such as reactive oxygen species, pH, O_2_, immunosuppressive cells, cytokines, and metabolites, as well as those that impair cell trafficking into the tumor microenvironment. Next-generation CAR-T cell therapy is currently undergoing clinical trials to overcome these challenges. Therefore, novel approaches to address the challenges faced by CAR-T cell immunotherapy in solid tumors are also discussed here.

## 1. Introduction

For a long time, cancers have been treated using traditional therapies, such as surgery, radiation therapy, and chemotherapy. Although these therapies are still popular, as they have considerable effects in terms of prolonged survival, they also have limitations and severe side effects. Recently, targeted cancer therapies, like imatinib and trastuzumab [1], which interfere with the activity of specific molecules related to cell proliferation, have also been developed and applied as standard therapies for many cancers. More recently, immunotherapy, which boosts and strengthens a patient’s own immunity to control tumors, has emerged and paved the way for a new era of cancer treatment, leading not only to prolonged survival, but also to total recovery. Chimeric antigen receptor (CAR) T cells, as a rapidly emerging immunotherapeutic modality, are T cells that are genetically engineered to express an antigen-specific receptor that can recognize a target in a non-MHC restricted manner, unlike conventional T cell receptors (TCRs) [2].

CAR-T cell therapy has provided a dramatically advanced breakthrough as one of the most promising cancer immunotherapies [3]. Despite the advances in CAR-T cell therapy for hematologic malignancies, its use for solid tumors remains challenging because of issues involving on-target/off-tumor activity and anatomical and environmental features. One of the main reasons for CAR-T cell therapy failure in solid tumors is the unavailability of solid tumor-specific antigens, unlike in chronic lymphoblast leukemia (CLL) and acute lymphoblast leukemia (ALL), which universally express the antigen CD19 on B cells [4]. Tumor antigens are mainly classified into two categories: (i) tumor-specific antigens (TSAs), which are specifically expressed on tumor cells and can thus be targeted with fewer side effects (such as on-target/off-tumor toxicity); and (ii) tumor-associated antigens (TAAs), which are expressed on cancer cells, as well as healthy cells (often in lesser quantity), and are highly prone to causing excessive toxicity upon being targeted [5]. As solid tumors scarcely express one TSA, TAA or a combination of TAAs are commonly targeted for immunotherapies against most solid tumors [6].

**Figure 1:**
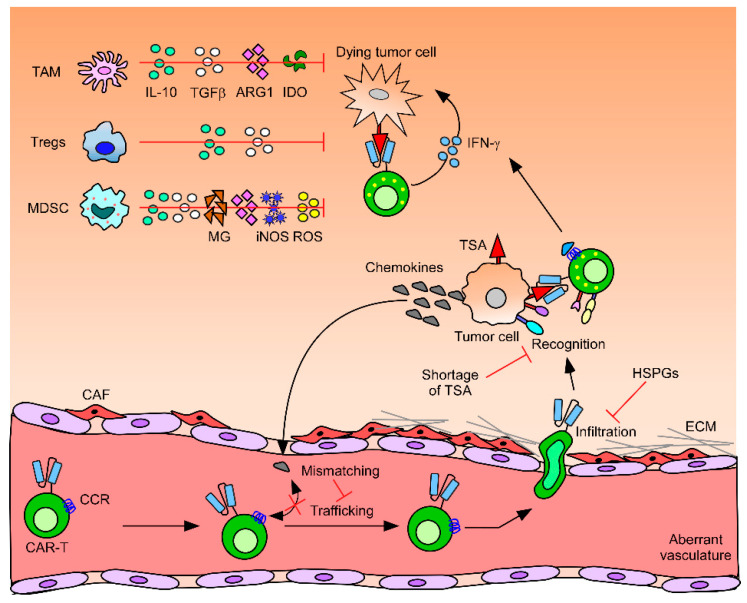
The journey of chimeric antigen receptor T (CAR-T) cell from the bloodstream to the tumor microenvironment and the immunosuppressive challenges it faces. A CAR-T cell starts its journey in the bloodstream, which is the common site of administration. It faces challenges regarding infiltration because of the lack of cognate chemokine signaling, aberrant vasculature, and extracellular matrix (ECM) proteins, such as heparan sulfate proteoglycans (HSPGs). Eventually, after infiltration, it encounters complications in recognizing tumors because of the shortage of TSA. It further faces an inhibitory environment because of soluble immunosuppressive factors produced by tumor-associated macrophages (TAMs), regulatory T cells (Tregs), and myeloid-derived suppressor cells (MDSCs), and its cytotoxic efficacy is thus attenuated. The factors that interfere with the effective anti-tumor response of CAR-T cells are controllable, either individually or in combination, to improve CAR-T cell infiltration, persistence, and cytotoxicity. CCR, cognate chemokine receptor; TSA, tumor-specific antigen; IL, interleukin; TGFβ, transforming growth factor-β; IDO, indoleamine-2,3-dioxygenase; CAF, cancer-associated fibroblast; ROS, reactive oxygen species; MG, methylglyoxal; iNOS, inducible nitric oxide synthase.

The tumor microenvironment (TME) in solid tumors is less accessible and immunosuppressive. The TME is redesigned by cancer cells to facilitate their growth and is not a favorable environment for T cell homing or persistence [5]. For a clinically useful anti-tumor response, CAR-T cells need to overcome several obstacles, such as insufficient infiltration, mismatched chemokine signals, physiological barriers, immunosuppressive cytokines/cells, pH, oxidative stress, immune checkpoint molecules, antigen escape, and scarcity of immune-stimulating cytokines [7]. These immune invasion factors hinder CAR-T cell function, as illustrated in Figure 1. Additionally, mechanisms for CAR-T cell resistance are rapidly emerging [8]. Because the natural machinery of T cells is not sufficient to overcome the severe challenges mentioned above, many studies have been performed and many are currently underway to artificially modify these cells to allow them to infiltrate, persist, and proliferate in and attack tumors. In this review, we discuss the limitations of CAR-T cell therapy in solid tumors and the advanced strategies that are currently being tested to overcome these limitations. Limiting factors identified in different solid tumor models and the corresponding studies are summarized in Table 1.

## 2. Overview of CAR-T Cells

CAR-T cells contain a CAR that is capable of recognizing antigens independent of an MHC molecule. The first-generation CARs contained a single-chain variable fragment (scFv) of an antibody fused to a transmembrane domain and an intracellular CD3ζ signaling domain that induces T cell activation [9]. Later, additional co-stimulatory domains, like CD28, 4-1BB, CD27, ICOS, and/or OX40, were added to the CAR individually or in combination (called second-/third-generation CAR-T cells) and have resulted in better persistence, robust activation, and enhanced anti-tumor activity [10]. CARs are mainly transduced by viral methods, with lentiviral transduction being the most common method [11]. Other non-viral transduction methods have emerged that utilize the DNA or mRNA transposon systems and have proved to be less mutagenic [12]. Further research into overcoming a suppressive microenvironment has led to the development of CAR-T cells that have cytokine transgenes, and can thus modulate cytokine function or release cytokines [2,13,14,15]. In 2017, the U.S. Food and Drug Administration (FDA) approved two CAR-T cell therapies, KYMRIAH^®^ (tisagenlecleucel) and YESCARTA^®^ (axicabtagene ciloleucel), for pediatric ALL and adult relapsed/refractory large B cell lymphoma, respectively, which achieved complete remission in 90% of cases. These CAR-T cell therapies were approved by the European Medical Agency (EMA) in 2018 [16,17]. However, despite extensive research, CAR-T cell therapy has been scarcely successful in solid tumors.

## 3. Limiting Factors for CAR-T Cell Therapy of Solid Tumors

### 3.1. CAR-T Cell Trafficking to the Tumor Site

Solid tumors can undergo immune escape by interfering with T cell trafficking. Furthermore, T cell migration is closely correlated with the prognosis of patients with various cancers, including thymoma, mesothelioma, brain, ovarian, pancreatic, and skin cancers [18,19,20,21]. Improved T cell trafficking to a tumor site has been shown to be positively correlated with enhanced anti-tumor responses [22]. Therefore, enhanced T cell trafficking to a tumor site is essential for the success of CAR-T therapy. The poor homing of CAR-T cells to a tumor site is caused by chemokine–chemokine receptor mismatching, downregulation of adhesion molecules, and abnormal tumor neovasculature, which exhibits irregular distribution, branching, and cellular composition (Figure 1) [22]. Indeed, although some solid tumors highly express C-C chemokine ligand 2 (CCL2), cognate chemokine receptors (CCRs), such as CCR2b and CCR4, are rarely expressed on T cells, resulting in poor homing to the solid tumor bed [23]. Expression of the adhesion molecules Intercellular Adhesion Molecule (ICAM)-1/2, Vascular Cell Adhesion Molecule (VCAM)-1, and CD34 is downregulated on some tumor endothelia to recruit suppressive cells and exclude effector T cells [24]. In terms of aberrant vascular morphology, pericytes, which are a critical vascular cell type essential for vessel maintenance and remodeling, are scarce or loosely attached to tumor blood vessels, resulting in leakier vessels than normal [25]. These leaky blood vessels induce aberrant blood flow, limiting T cell migration to the tumor site [26].

### 3.2. CAR-T Cell Infiltration into the Tumor Bed

Once T cells successfully traffic to tumor sites, T cell infiltration into the dense tumor cell region is required. However, in various solid tumors, T cells are physically trapped and prevented from penetrating the tumor by the dense extracellular matrix (ECM) produced by cancer-associated fibroblasts (CAFs) [27,28,29,30,31,32,33,34,35]. Additionally, CXCL12, produced by CAF, protects cancer cells from anti-tumor T cells. The generation of blood vessels, known as high endothelial venules (HEVs), is critical for CAR-T cell infiltration and is associated with tumor regression in tumors, such as melanoma [36]. The ECM, containing proteoglycans and glycopeptides, is composed of nonstructural matrix proteins, such as heparan sulfate proteoglycans (HSPGs), and has an essential role in the tumor cell growth [37,38]. T cell penetration and function in stroma-rich solid tumors are significantly impaired by the abundance of ECM (Figure 1), resulting in the lower killing activity of CAR-T cells [39]. Indeed, recent studies showed that the expression of heparanase (HPSE), which can degrade HSPGs, is reduced in in vitro developed CAR-T cells [40].

### 3.3. Immunosuppressive Tumor Microenvironment 

#### 3.3.1. Immune Suppressor Cells

The suppression of the anti-tumor immune response of CAR-T cells in solid tumors is mediated by immunosuppressive cells, including regulatory T cells (Tregs), myeloid-derived suppressor cells (MDSCs), and TAMs, in the TME [41,42,43,44]. These cells release cytokines, such as transforming growth factor-β (TGFβ) and IL-10, resulting in the suppression of the cytotoxic effect of CAR-T cells. MDSCs, which are representative immunosuppressive cells, not only regulate the anti-tumor activity of CAR-T cells negatively, but are also involved in tumor progression by expediting tumor angiogenesis and tumor cell invasion. MDSCs suppress T cell proliferation and function through arginase, inducible nitric oxide synthase (iNOS), TGFβ, and IL-10 [45]. Moreover, MDSCs induce the production of reactive oxygen species (ROS), resulting in a robust immunosuppressive microenvironment (Figure 1). Therefore, inhibition or depletion of MDSCs has improved anti-tumor immune response in the TME. Furthermore, MDSCs in the TME recruit Tregs, which play a critical role in the negative regulation of the immune response. Moreover, these cells suppress the T cell activity through various mechanisms, including cell-to-cell interaction, inhibition, and secretion of immunosuppressive cytokines, such as TGFβ and IL-10 [46]. Most tumor cells induce immunosuppressive M2 macrophages rather than immunostimulatory M1 macrophages. TAMs promote the proliferation, activation, and metastasis of tumor cells by expressing various immunosuppressive soluble factors [47].

#### 3.3.2. Immune Checkpoints

Tumor-infiltrating T cells are functionally tolerant to TME and display suppressed effector functions compared with those of normal effector T cells [48]. They express inhibitory receptors, such as PD-1, TIM-3, TIGIT, LAG-3, and CTLA-4 [49,50,51], which induce T cell exhaustion and prevent the excessive activation of T cells; however, cancer cells exploit these receptors to evade the attack of T cells [52,53,54]. PD-1 regulates T cell activation via binding to PD-L1 [55] or PD-L2 [56] on the surface of tumor cells [57]. PD-1/PD-L1/2 binding inhibits T cell proliferation and IFN-γ, TNF-α, and IL-2 production and limits T cell survival [58]. Similar to PD-1 signaling, CTLA-4 binds to B7 and inhibits CAR-T cell activation directly at the TCR immune synapse [59,60].

High expression of immune checkpoint proteins on CAR-T cells is associated with disrupted effector function, loss of tumor immune response, and poor prognosis [61]. Higher expression of ligand for immune checkpoints on tumor cells is associated with poor anti-tumor response and poor prognosis. In addition to PD-1 and CTLA-4, various co-inhibitory receptors, such as TIM-3, LAG-3, and TIGIT, are also expressed on exhausted T cells [62]. Thus, consecutive targeting of immune checkpoint signaling pathways may further enhance CAR-T cell potency.

#### 3.3.3. Reactive Oxygen Species (ROS) 

The TME of solid tumors suppresses the function of infiltrated T cells via several mechanisms, such as ROS generation [63,64]. Infiltrated T cells often encounter abundant oxidative stress that can attenuate their anti-tumor effects. The accumulation of ROS in TME is induced by MDSCs. MDSC-released ROS modifies TCR and CD8 or downregulates CD3ζ expression, thereby inhibiting the antigen-specific response [65] and activity of effector T cells [66]. MDSCs are present in most solid tumors and can produce arginase, which deprives T cells of amino acids and suppresses the T cell response, with decreased IL-2 and IFN-γ expression [67,68]. Excessive ROS is produced in TME because of an imbalance between ROS production (mainly from mitochondria through respiratory burst) and detoxification. While the normal ROS levels are essential for cell signaling, an abrupt increase in ROS levels, primarily owing to environmental stressors, such as pollutants and ionizing radiation, causes cell damage and carcinogenesis [69]. Oxidative stress is known to cause cell injury through mutations, especially in cell cycle-related genes and the tumor suppressor gene *p53*, which leads to tumorigenesis and tumor progression [70].

#### 3.3.4. Metabolites

The T cell-mediated anti-tumor response requires a balance of nutrients and energy for T cell expansion and function. However, tumor cells have high metabolic demands, resulting in an inhibited function of CAR-T cells in the TME. In a murine tumor model, the reduction of metabolites inhibited MDSC-mediated T cell suppression and improved the anti-tumor activity [71]. Thus, controlling metabolites in the TME is important. Lactate, one of the metabolites of tumor cells, suppresses the proliferation, activation, cytokine production, and cytotoxicity of T cells [72]. Other metabolites of tumor cells, including adenosine [73], indoleamine-2,3-dioxygenase (IDO) [74], arginase-1 [75], and L-arginine [76], are amino acid-degrading enzymes that are commonly expressed in the TME and also inhibit T cell function [77,78,79]. Recently, Baumann et al. reported that MDSC-dependent metabolites constrain the anti-tumor response of CD8^+^ T cells via the cell-to-cell transfer of the toxic metabolite methylglyoxal (MG) to the cytosol of T cells [71]. The MG-responsible factors and MG-linked pathway in T cells are unclear. Previous studies have suggested that MG is linked with the oxidative stress response involving Nuclear Factor Erythroid 2-Related Factor 2 (NRF2) [80] and the Hippo signaling pathway [81]. Taken together, the optimal balance between T cell and tumor cell metabolism is essential for tumor control. Modulation of metabolites improves CAR-T cell efficacy, including promotion of the generation of central memory-like cells and reduction of T cell death [82].

#### 3.3.5. Cytokines

Tumor-derived cytokines, related to the inflammatory response at tumor sites, may attenuate the anti-tumor response of CAR-T cells, which is suppressed because of immunosuppressive cytokines in various solid tumors [83,84,85,86,87]. At tumor sites, immunosuppressive cytokines, such as TGFβ, IL-10, and IL-6, are produced by immunosuppressive cells [88,89]. These play a pivotal role in the T cells’ anti-tumor response. One of the most critical inhibitory tumor-associated cytokines is TGFβ [90,91]. TGFβ suppresses CD8^+^ effector T cell function and induces Treg maturation. Inhibiting TGFβ by an antibody improves CD8^+^ T cell effector function and promotes the anti-tumor response by elevating CAR-T persistence and infiltration into the tumor [92]. The expression of IL-10 inhibits the activation of T cells and natural killer (NK) cells, resulting in the proliferation and activation of tumor cells [93]. Additionally, granulocyte-macrophage colony-stimulating factor (GM-CSF), which is an M2 immunosuppressive soluble protein, induces the proliferation, invasion, and metastasis of tumor cells [94].

#### 3.3.6. pH

Oxidative stress is accompanied explicitly by an acidic microenvironment. This acidic condition promotes tumor progression and metastasis. Many regions in a tumor mass are acidic. In solid tumor cells, glucose metabolism is increased, resulting in a significant generation of lactate and H^+^ [72,95]. Acid produced in tumor cells is transported out of the cells, making the extracellular space acidic. Tumor cells are well adapted to the acidic microenvironment, as shown through in vitro studies, such that the optimal proliferation of tumor cells occurs at a pH of 6.8 instead of the pH of 7.3, which is required by healthy cells [96]. A low pH triggers the formation of a fibrous-structure around the tumor and restricts the regular movement and migration of T cells [97]. Therefore, T cells fail to respond against tumor cells (Table 1) [98].

#### 3.3.7. Hypoxia 

A poor tumor microenvironment has low oxygen (O_2_) pressure [113]. Tumor infiltrating T cells undergo anergy, have dysfunctional cytotoxicity, grant cancer-therapy resistance to the tumor, as well as lead to a more malignant phenotype [100,114]. Recent studies have reported that CAR-T cell designs that have oxygen-sensing factors, such as HIF-1α, result in the promotion of memory-associated metabolic pathways, such as fatty acid oxidation, and improve their function in a hostile microenvironment [35,79,115]. Therefore, tumor infiltrating T cells are stabilized in response to hypoxia and are restricted to the local tumor environment, minimizing their potential “on-target/off-tumor” effects [79].

### 3.4. Shortage of Tumor Antigens

The major difference between solid tumors and hematological tumors is that it is more challenging to find TSAs for solid tumors. Many studies have employed immunoproteomics to discover TSAs against the immunogenic antigens of tumor cells. For most solid tumors, it is common to find TAAs, which are expressed at lower levels on cells of healthy tissues than on tumor cells. The representative TAAs for solid tumors are carcinoembryonic antigen (CEA); v-erb-b2 avian erythroblastic leukemia viral oncogene homolog 2, also known as HER2 (ERBB2); epidermal growth factor receptor (EGFR); EGFR variant III (EGFRvIII); glypican 3 (GPC3); epithelial cell adhesion molecule (EpCAM); ganglioside G2 (GD2); mesothelin; cell surface-associated mucin1 (MUC1); and prostate stem cell antigen (PSCA) [85,86,100]. The shortage of TSA severely limits the use of CAR-T therapy for solid tumors and increases the on-target/off-tumor toxicity [6]. Therefore, dual CARs targeting a combination of tumor antigens have been developed to address both antigen heterogeneity and the threat of antigen loss [100]. Therefore, the targeting of multiple-tumor-antigen by CAR-T cells improves their effector function and anti-tumor activity.

## 4. Approach to Improve CAR-T Cell Therapy for Solid Tumors

### 4.1. CAR-T Cell Trafficking and Infiltration

In contrast to the case of blood malignancies, infiltration is a limitation for CAR-T cell therapy in solid tumors. The tumor mass has a certain histopathology that is rich in blood vessels, fibroblasts, and myeloid cells, which safeguards tumor cells and limits CAR-T cell infiltration because of anatomical and physiological reasons [116]. One of the preferred approaches for handling this issue is the regional delivery of CAR-T cells. This is possible in most tumor models via intratumoral delivery. This approach has been tested using mesothelin-expressing CAR-T cells directed at malignant pleural cancers. Intrapleural administration has shown enhanced activation and persistence compared with the intravenous injection of CAR-T cells in the same model; its advantages include low systemic toxicity and the requirement of few cells to produce the same therapeutic effect [117]. Local delivery has also been tested in head and neck cancers and glioma models through intracranial/intra-ventricular administration, which provided better results [117,118,119]. Additionally, instead of injecting CAR-T cells into the bolus, the cells can initially be absorbed into a biopolymer along with other co-activating molecules. The biopolymer scaffold can then be inserted near the tumor mass, where it can release CAR-T cells at an optimal concentration and at a constant rate [120].

Chemokines are vital molecules that facilitate the infiltration of T cells into a tumor mass. They bind with the corresponding receptors on T cells, leading to the activation of intracellular signaling mechanisms and the subsequent polarization of cells migrating toward the higher concentration of chemokines [121]. In vivo, chemokines are expressed by various cells, including stromal cells, innate immune cells, and even tumor cells [122,123,124]. The chemokines expressed in the TME do not often match the receptors expressed on T cells. This chemokine–chemokine receptor mismatch is one of the most widely studied and immensely exploited mechanisms of insufficient T cell infiltration and subsequent tumor immune evasion [125].

**Figure 2:**
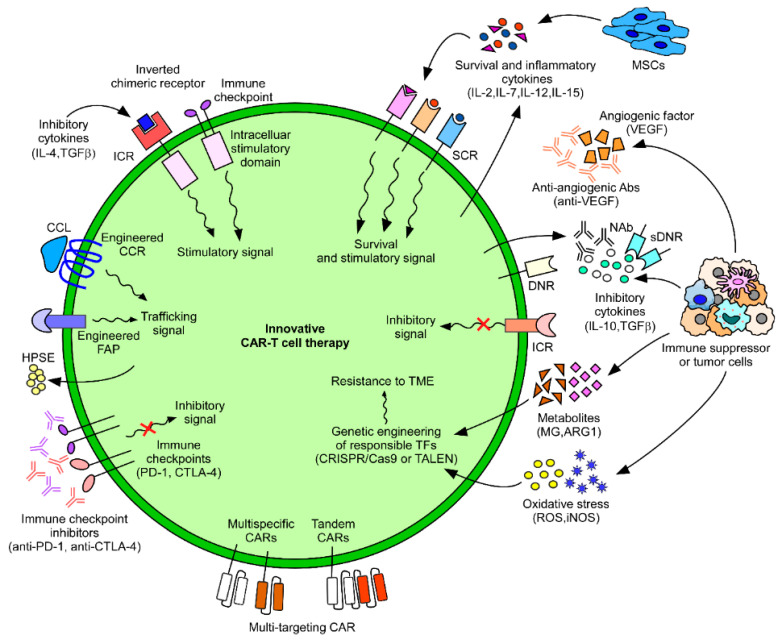
Approaches for improved CAR-T cell therapy. Innovative approaches of CAR-T cell therapy for solid tumors. Inverted chimeric receptors convert the inhibitory signals from cytokines (IL-4 or TGFβ) or immune checkpoint to stimulatory one via intracellular stimulatory domain. Engineered expression of cognate chemokine receptors (CCRs) with matching of chemokines (CCL), fibroblast activated protein (FAP), and heparanase (HPSE) induces trafficking signals and T cell infiltration into tumor microenvironment (TME). As immune checkpoints, such as PD-1 or CTLA-4, suppress T cell activation, blocking their signals with immune checkpoint inhibitors (anti-PD-1 or anti-CTLA-4) enhances CAR-T cell cytotoxicity. Multi-specific CARs and tandem CARs target multiple tumor antigens to boost its function. Genetic engineering of responsible transcription factors (TFs) to regulatory signals of oxidative stress (ROS and iNOS) and immunosuppressive metabolites (MG and ARG1), from either tumor cells or immune suppressor cells, provides CAR-T cells the resistance to TME. NAbs or sDNR against inhibitory cytokines, such as IL-10 and TGFβ, prevent inhibitory signals via inhibitory cytokine receptors (ICR). Anti-angiogenic antibodies (anti-vascular endothelial growth factor (VEGF)) block angiogenesis and then improve T cell infiltration into the tumor bed. MSCs or CAR-T cells engineered to express survival or inflammatory cytokines, IL-2, IL-12, IL-7, and IL-15, enhance T cells function and maintenance. Combinations of these approaches in a solid tumor models enhance T cell cytotoxicity. CR: cytokine receptor, iNOS: inducible nitric oxide synthase, sDNR: soluble dominant negative receptors, NAb: neutralizing antibody, ICR; inhibitory cytokine receptor, SCR: stimulatory cytokine receptor, MSCs: mesenchymal stem cells.

Several studies have exploited this phenomenon and have mainly engineered cognate chemokine receptor on CAR-T cells (Figure 2). These experiments started in 2002, when Kershaw et al. engineered a CXCR2 receptor on T cells for its cognate cytokine CXCL1, and this led to the enhanced chemotactic behavior of T cells [126]. Later, Di et al. engineered a chemokine receptor CCR4 on adoptively transferred CAR-T cells intended to be paired with thymus- and activation-regulated chemokine/CC chemokine ligand 17 (TARC/CCL17) on CD30 positive cells in a Hodgkin lymphoma mouse model [127]. Similarly, in another study, CCR2 was engineered on CAR-T cells for targeting its cognate chemokine CCL2, which is highly expressed in malignant pleural mesothelioma. The engineered CAR-T cells exhibited increased migration and a 12.5-fold increase in cytotoxicity compared with non-transduced CAR-T cells [21]. CCR2b transduction in GD2 (a glycolipid antigen)-expressing CAR-T cells was also studied in a neuroblastoma model. A previous study demonstrated a 10% enhanced homing in vitro and enhanced anti-tumor activity in vivo [19].

CCL17 and CCL22 are cytokines expressed by stromal cells in the TME and are responsible for the homing of immune regulatory Treg and Th2 cells, which express their cognate receptor CCR4. Additionally, because of mutations in *GATA3*, CCR4 is also expressed in lymphoma cells, which leads to the further recruitment of malignant cells. This results in an overall inhibitory profile and has pro-tumoral effects. An effective strategy is to engineer CCR4 on CAR-T cells that can lead to anti-tumor effects. This receptor is also an effective target for monoclonal antibodies. A CAR-T cell against CCR4 was developed in 2007 [128]. Mogamulizumab is a monoclonal antibody against CCR4 that was designed in Japan and licensed for the management of relapsed or refractory adult T cell leukemia/lymphoma in 2012 and elapsed/refractory CCR4^+^ cutaneous T cell lymphoma in 2014 [129].

Other studies have aimed to enhance CAR-T cell infiltration by focusing on antigens present in tumor surroundings. For example, CAR-T cells targeting the vascular endothelial growth factor receptor-2 (VEGF-R2), an antigen expressed on the tumor vasculature, hinder tumorigenesis by blocking angiogenesis, and thus facilitating tumor infiltration [6]. In addition, anti-angiogenic antibodies have also been shown to exhibit enhanced T cell infiltration into the tumor bed (Figure 2) [39].

Another study targeted fibroblast activation protein (FAP), which is expressed on tumor stromal cells. CAR-T cells targeting FAP blocked stroma formation (stromagenesis), thereby decreasing tumorigenesis and enhancing infiltration [130,131]. An echistatin-containing CAR (eCAR) that has a high affinity for the αvβ3 integrin, physiologically expressed on endothelial cells and pathologically expressed on tumor cells, leads to a decrease in tumor size [132,133]. Additionally, CAR-T cells genetically engineered to express HPSE that can degrade HSPGs have a higher anti-tumor efficacy [40].

### 4.2. CAR-T Cell Resistance to the TME

Once CAR-T cells reach the TME, they face the challenges of persistence and expansion. As the TME is not a suitable environment for T cells, many immune inhibitory factors (mentioned above) limit the efficacy of CAR-T cells (Figure 1). Many limiting factors have been exploited by scientists to facilitate T cell growth and activity. 

TME immunosuppression mainly occurs via immunosuppressive cytokines, immunosuppressive cells, and the lack of immune-activating factors. One practical approach is changing the immunosuppressive environment into an activating one by engineering chimeric inverted receptors (Figure 2), such as IL-4, secreted by tumors that have an inhibitory effect on T cells. An inverted cytokine receptor was engineered on T cells, which had the exodomain of IL-4 and the endodomain of IL-7; the results showed that the inverted cytokine receptor could convert suppressive signals into T cell proliferative signals in prostate stem cell antigen (PSCA)-specific CAR-T cells and caused enhanced anti-tumor immunity in prostate cancer models [134]. Another group used a similar approach and constructed a chimeric PD-1 receptor with the truncated extracellular domain of PD-1 and transmembrane and internal domain of CD28, thus converting the PD-1 signal into a stimulatory one [135]. This approach has also been tested for CTLA-4- and TGFβ-mediated inhibition [136,137].

Other studies focused on neutralizing immune suppression and employed various strategies (Figure 2), like the administration of neutralizing antibodies against IL-10, along with specific CAR-T cells, which caused enhanced T cell proliferation [138]. TGFβ, an inhibitory cytokine, has been neutralized using a dominant-negative receptor (DNR) [139,140,141]. Some studies reported neutralization by engineering T cells to secrete inhibitors, for example, CAR-T cells engineered to secrete PD-1 inhibitors are currently being tested [142]. PD-1 neutralization has also been studied using CAR-T cells with PD-1-blocking monoclonal antibodies (mAbs) [143]. Additionally, the inactivation of the PD-1 receptor, by internal disruption (knockouts) using the CRISPR/Cas9 system [144] or via transcription activator-like effector nucleases (TALENs) [145], makes CAR-T cells resistant to PD-1-mediated inhibition. Another study attempted to generate immune suppression resistant CAR-T cells by knocking out both PD-1 and CTLA-4 using the CRISPR/Cas9 system [144]. Combining CAR-T cells with mAbs for immune checkpoints, such as TIM-3 and LAG-3, is another important strategy. A bispecific antibody targeting PD-L1 and LAG-3 also enhances anti-tumor activity [146]. All of the above-mentioned factors (PD-1, TIM-3, and LAG-3) are markers for exhaustion, which render T cells ineffective, mostly because of chronic stimulation [49]. Lynn et al. showed that the transcription factor c-Jun (part of the canonical AP-1 c-Fos–c-Jun heterodimer) plays a critical role in preventing adoptively transferred CAR-T cell exhaustion and that the efficacy of CAR-T cell therapy is significantly enhanced by c-Jun overexpression in several models [147].

Besides neutralizing immune-suppressive signals, CAR-T cells can also be engineered to enhance immune-stimulating effects by secreting stimulatory cytokines (Figure 2). IL-2 and IL-12 enhance the proliferation of T cells and have a regulatory effect on Tregs and MDSCs. T cells engineered to secrete the cytokines IL-12 [15], IL-15 [148], IL-18 [13], and IL-21 [14] have led to an enhanced pro-inflammatory effect. Local delivery of IL-12 with VEGFR-2-targeting CAR-T cells led to increased survival in mice with subcutaneous tumors [100]. CAR-T cells expressing IL-7 (for enhanced proliferation and survival) and CCL19 (for chemoattraction), called 7 × 19 CAR-T cells, enhanced anti-tumor activities and increased the infiltration of dendritic cells and T cells in the TME [149]. Engineering the receptors of immune-stimulatory cytokines also led to enhanced signaling. For example, engineering the IL-7 receptor on CAR-T cells has shown enhanced anti-tumor activity in triple-negative breast cancer (TNBC) [150], and engineering the IL-15 receptor [151], improving its local delivery through armed oncolytic virus [152], has also shown enhanced anti-tumor activity. Furthermore, substituting IL-7 or IL-15 for IL-2 during the ex vivo expansion of CAR-T cells enhanced the T memory stem cell phenotype, reduced exhaustion markers, and improved proliferation and anti-apoptotic functions [153,154].

Other TME immunosuppressive factors include hypoxia, immunosuppressive cells, and competition among immune cells for the key nutrient glucose and for amino acids [107]. One approach for CAR-T cell design is to ensure more activity in the hypoxic microenvironment; this was achieved by one group, by fusing CAR to oxygen-sensitive HIF-1α [79]. Other studies have focused on chemotherapy-mediated lymphodepletion in blood malignancies and solid tumors [155,156]. This enhances CAR-T cell expansion through the depletion of inhibitory immune cells and less metabolic competition and also provides “space” for CAR-T cells to grow [157].

We have discussed various approaches to overcome the inhibitory TME through modification of CAR-T cells. Another recent significant approach is the use of mesenchymal stem cells (MSCs) that are capable of homing to the tumor tissue to modulate the immunosuppressive TME (Figure 2). Taking advantage of the inherent tumor-homing ability of MSCs, MSCs engineered to produce both IL-7 and IL-12, which are critical in the survival and protective responses of T cells, converted from the inhibitory to the stimulatory TME and enhanced the maintenance and anti-tumor activity of CAR-T cells [158]. Thus, this implicated that MSCs can be applied as vehicles for the TME modification.

### 4.3. Multi-Targeting CAR-T Cells

Finding an appropriate antigen that is explicitly expressed on tumor cells is one of the important challenges of CAR-T cell development. Many currently tested CAR-T cells for solid tumors target TAAs, which are also present in normal cells and have the potential to cause on-target/off-tumor toxicity upon being targeted [159]. Additionally, one of the many mechanisms involved in tumors developing resistance to CAR-T cells is antigen escape [160]. Antigen escape includes antigen downregulation, antigen decrease, and/or complete loss of antigen density. For FDA-approved CD19 CAR-T cells, the relapse rate is 30−60%, which is mainly attributed to antigen loss and insufficient CAR-T cell persistence [82], and has been proven by the growth of CD19 negative leukemic cells [161]. CARs targeting multiple antigens are currently being tested to overcome this situation by helping in the differentiation between tumors and healthy cells. Strategies mainly include using Boolean logic gates, which comprise AND, OR, and NOT gates. CAR-T cells with an AND gate get activated only in the presence of both pre-determined antigens, those with an OR gate require either of the two pre-determined antigens, whereas those with the NOT gate help identify normal cells through deactivation if a specific antigen is detected [162]. Many combination strategies are currently being applied, which include pooled, bispecific/trispecific, and tandem CARs (Figure 2). The simplest of all is called cocktail immunotherapy, which includes pooled CAR-T cells; it uses two different CAR-T cells, each targeting a single antigen, administered together, and mainly using the OR gate. This strategy helps in targeting tumor cells even when one antigen has escaped or its density is reduced. Sequential, instead of pooled therapy, has also shown efficacy. Most commonly, CD123 paired with CD19 has shown positive results in combating CD19-mediated antigen escape and resistance [163]. Additionally, EGFR- and CD133-specific CAR-T cells have shown positive results in cholangiocarcinoma [164]. Bispecific (bivalent) and trispecific (trivalent) CAR-T cells utilize two or three different CAR receptors expressed in a single T cell. They can use all the logic gates and are designed based on the cancer type. Because they have a comparatively higher density of CARs, they demonstrate longer persistence and higher anti-tumor activity [163,165]. In contrast, tandem CAR-T cells have two different scFv regions within one CAR in a single T cell, which targets two antigens [166] and can have different gating types according to the tumor type. To enhance functional CAR-T cell persistence and improve the therapeutic efficacy of CAR-T cells in solid tumors, Ma et al. reported a unique strategy using amphiphile CAR-T ligands (amph-ligands), which are transferred to the lymph nodes (LNs) upon inoculation and, adoptively, into the amph-ligand-primed native LN of mice, resulting in the induction of CAR-T cell expansion and an enhanced anti-tumor response in multiple mouse tumor models [167].

Apart from the gates and pooling strategies mentioned above, many other approaches have been employed, including switchable CAR-T cells that can be activated only in the presence of an internal (e.g., a TME factor) or external (often a small molecule) switch [168]. Some CAR designs use a peptide that blocks the scFv region until it is cleaved by factors in the TME, thus inactivating CARs until the CAR-T cells reach the tumor site [169]. A SynchNotch receptor, on the contrary, when activated by its cognate antigen, is translocated to the nucleus to induce CAR expression [170]. A CAR can also be administered with an antibody that acts as a bridge between the CAR and the antigen [171].

Recent studies have used CAR-T cell therapy for cancer stem cells (CSCs). Because these cells have a different phenotype than other cancer cells and are responsible for most tumor proliferation, their targeting can be an effective strategy for tumor therapy [172]. Although relevant studies are in the initial stages, CD133 is commonly used as a target for CSCs [173]. A phase 1 clinical trial successfully showed the elimination of a CD133^+^ tumor, but CD133^−^ tumor relapse was observed [174]. More recently, KIAA1114 has been revealed as a distinct and stable marker for CSCs in hepatocellular carcinoma, and, Kiatomab, a mAb specific to KIAA1114, showed therapeutic potential in a murine tumor model, suggesting that KIAA1114 could be applied as a novel target antigen for CSC CAR-T cell therapy [175].

In an effort to develop novel checkpoint regulators, Hombach et al. reported the possibility of targeting the CD30–CD30L interaction [165]. CD30 and/or CD30L is expressed on T cells, B cells, and some APCs, and transiently upregulated upon activation. Moreover, previous reports have suggested that it may play a negative role in T cell activation. Thus, while CD30 was previously shown to play a role as a tumor antigen in hematological malignancies, it may also serve as a powerful immune checkpoint target. In a recent publication, Hombach et al. designed novel bispecific CAR-T cells that block the CD30 interaction and target carcinoembryonic antigen (CEA) and showed that bispecific CAR-T cells rejected CEA^+^ tumors more effectively than control CEA CAR-T cells [165].

Although multi-antigen-targeting CAR-T cells are effective for addressing antigen escape, they are still vulnerable to inactivation by checkpoint inhibitors, lesser infiltration, and persistence, like all other CAR-T cell types. Research focusing on multi-antigen-targeted CAR-T cells is still in its initial phase involving in vivo studies, and many adverse effects of CAR-T cells, such as cytokine release syndrome, need to be effectively tested [162].

## 5. Conclusions

Cancer therapy has continually evolved over the past several decades. After chemotherapy, radiotherapy, and surgery, we are in the era of immune-targeted therapy, which has shown promising results for cancer therapy and is a way forward. Especially, CAR-T cell therapy has shown promising results in accurate tumor targeting. However, because of the scarcity of TSA, antigen loss, immunosuppressive TME, and many other factors, this mode of therapy is currently limited. Additionally, CAR-T cells pose severe risks of adverse effects, such as on-target/off-tumor toxicity, that have resulted in deaths and subsequent discontinuation of many clinical trials. An unprecedented number of preclinical studies and clinical trials are currently in progress for solid tumor therapies, targeting a plethora of antigens in various tumor models. Further studies focusing on overcoming the limitations of current strategies, exploring further molecular mechanisms, and tackling antigen escape are in progress. Many studies focus on making CAR-T cells resistant to the immunosuppressive TME, improving trafficking, removing physiological/anatomical barriers in migration, and targeting multiple antigens (Figure 2). In particular, the prospects for future approaches in terms of CAR-T cell survival and function can be summarized as follows. (1) Chimeric cytokine receptors and coreceptors; they greatly contribute to strengthening the killing effect of CAR-T cells by converting inhibitory signals into stimulatory signals. (2) Neutralization of inhibitory factors generated by the TME; antibodies and soluble receptors are used to improve CAR-T survival and activity. (3) CAR-T cells or MSCs engineered to produce pro-inflammatory and survival-related cytokines; the TME can be modified for prolonged and improved anti-tumor CAR-T cell responses through the CRISPR/Cas9 and TALEN system or transgenic technology. The combined application of these approaches will greatly contribute to maximizing CAR-T survival and anti-tumor function.

An important emerging technology involved CAR natural killer (NK) cells, which are challenging CAR-T cells in clinical trials in recent years. NK cells have an advantage over T cells in many ways. They do not require strict HLA matching, and thus avoid causing graft versus host disease (GVHD) [176]. Furthermore, CAR-NK cells, unlike CAR-T cells, retain their intrinsic cytotoxic ability; thus, they can lyse targeted cells independent of CAR-antigen interactions. CAR-NK cells have lesser toxicity because they do not pose the risk of cytokine release syndrome. Although it has many advantages, the application of CAR-NK cells for cancer therapy has many drawbacks as well. These include less efficient trafficking, immunosuppression by TME, inability to expand in vivo, less frequency of NK cells in the body as compared with that of T cells, deactivation in the freeze–thaw process, and inefficient transduction techniques [177]. These limitations render CAR-NK cells a suboptimal option as compared with CAR-T cells. Recent technologies, such as the use of the sleeping beauty transposon systems, have given hope for the development of better systems using CAR-NK cells. Additional preclinical and clinical studies are required to better understand CAR-NK cell biology and address its limitations [178]. Currently, ten clinical trials are being conducted on CAR-NK cells, which are registered at www.clinicaltrials.gov. These trials target both solid and blood malignancies. CAR-NK cells have been tested for tumor antigens (CD19, HER2, CD33, CD7, MUCI, and NR) and SARS-CoV-2 infected cells [179,180]. The number of clinical trials being conducted on CAR-NK cells is far lower that the number of clinical trials being conducted on CAR-T cells, which suggests that further research is required.

To bring CAR-T cells from bench to bedside, many factors still need to be considered. First, CAR-T cells remain an expensive option; this has led to research regarding universal CAR-T cells, but these have their drawbacks. CRISPR/Cas9 technology has transformed CAR, because it can knock out endogenous TCR and HLA, enhancing the antigen specificity of CAR-T cells and reducing their side effects. This can reduce the risk of GVHD, cross-reactivity, and rejection in the case of allogenic CAR-T cells. This technology can also knockout inhibitory receptors, such as PD-1, and can lead to the generation of off-the-shelf CAR-T cells with reduced manufacturing costs [181,182]. Other gene-editing technologies, such as TALENs, can also be used to produce similar results [183].

Moreover, further research into creating humanized scFv and fully humanized CAR-T cells is underway along with clinical trials, and is expected to result in better antigen recognition and few side effects [184]. CAR-T cell therapy involving CSCs is also a promising field, although current knowledge and studies regarding it are limited. Other research on combining CAR-T therapy with monoclonal antibodies, oncovaccines, and other small molecule inhibitors is underway to help scientists understand whether combination therapies can provide additive or synergistic effects. Another strategy can be to combine CAR-T cell or T cell therapy with factors that regulate T cell cytotoxicity through the modulation of cytokine signaling. We recently showed that the expression of soluble γc receptor (sγc) in T cells is highly upregulated upon TCR stimulation and inhibits the anti-tumor response of CD8^+^ T cells via regulating IL-2 and IL-15 signaling [185]. These results can be clinically translated for the effective adoptive T cell immunotherapy of cancers. Although CAR-T cells have not yet been approved by the FDA for solid tumor treatment and many further limitations need to be addressed, we have come a long way in engineering T cells and the TME for optimal anti-tumor effects. With each passing day, our understanding of cancer immunology, TSAs, TAAs, and TME is expanding, leading to further insights for better therapeutic options in the future.

## Figures and Tables

**Table 1:** Limiting factors for chimeric antigen receptor T (CAR-T) cell function in various cancer types.

	Disease	Brain Cancer	Breast Cancer	Cervical Cancer	Colorectal Cancer	Esophageal Cancer	Bone Cancer	Gastric Cancer	Liver Cancer	Lung Cancer	Lymphoma	Mesothelioma	Nasopharyngeal Cancer	Ovarian Cancer	Pancreatic Cancer	Prostate Cancer	Reanal Cell Carcinoma	Skin Cancer	Thymoma
Limiting Factor																			
Trafficking		✓ [19]										✓ [21]		✓ [21]	✓ [21]			✓ [20]	✓ [18]
Infiltration		✓ [32]	✓ [30,31]	✓ [34]	✓ [29]									✓ [27]	✓ [33]		✓ [28]	✓ [36]	
Immune suppressive TME	Immune suppressor cells								✓ [41]					✓ [42]				✓ [44]	
	Immune checkpoints	✓ [99]	✓ [99]			✓ [99]		✓ [99]		✓ [100]	✓ [99]			✓ [99]	✓ [99]	✓ [101]	✓ [99]	✓ [102]	✓ [99]
	ROS						✓ [103]			✓ [104]							✓ [105]		
	Metabolites		✓ [74]														✓ [100,105]		
	Cytokine								✓ [106]		✓ [83]			✓ [86]		✓ [85]		✓ [84]	✓ [87]
	pH		✓ [98]							✓ [98]				✓ [98]					
	Hypoxia									✓ [107]				✓ [42]				✓ [107]	
Shortage of tumor antigen (ClinicalTrials.gov) [108]	Mesothelin									✓		✓ [53]		✓	✓				
	EGFR	✓			✓														
	GPC3								✓										
	MUC1					✓				✓									
	HER2 (Human Epidermal Growth Factor Receptor 2)	✓	✓																
	GD2	✓																	
	CEA								✓						✓				
	EpCAM							✓	✓ [78]				✓						
	PSCA														✓				
Non-specific antigen On-target, off-tumor toxicity		✓ [109]								✓ [110]							✓ [111,112]	✓ [52,108]	

ROS, reactive oxygen species; TME, tumor microenvironment; EGFR, epidermal growth factor receptor; GPC3, glypican 3; MUC1, mucin 1 cell surface-associated; GD2, ganglioside G2; CEA, carcinoembryonic antigen; EpCAM, epithelial cell adhesion molecule; PSCA, prostate stem cell antigen.

## References

[1] Baker S.J., Reddy E.P. (2010). Targeted inhibition of kinases in cancer therapy. Mt. Sinai J. Med. A J. Transl. Pers. Med..

[2] Lepore M., Mori L., De Libero G. (2018). The conventional nature of non-MHC-Restricted T cells. Front. Immunol..

[3] Miliotou A., Papadopoulou L.C., Androulla M.N., Lefkothea P.C. (2018). CAR T-cell therapy: A new era in cancer immunotherapy. Curr. Pharm. Biotechnol..

[4] Orlando E., Leary R., Lacey S.F., Fraietta J., Bedoya F., Ambrose D., Wilcox N., Maude S.L., Frey N.V., Levine B.L. (2017). Gene expression signatures of response to anti-CD19 chimeric antigen receptor (CAR) T-cell therapy in patients with CLL and ALL. J. Clin. Oncol..

[5] Liu B., Yan L., Zhou M. (2019). Target selection of CAR T cell therapy in accordance with the TME for solid tumors. Am. J. Cancer Res..

[6] Ma S., Li X., Wang X., Cheng L., Li Z., Zhang C., Ye Z., Qian Q. (2019). Current progress in CAR-T cell therapy for solid tumors. Int. J. Biol. Sci..

[7] Stern L.A., Jonsson V.D., Priceman S. (2020). CAR T Cell Therapy progress and challenges for solid tumors. Infect. Complicat. Cancer Patients.

[8] Shah N.N., Fry T.J. (2019). Mechanisms of resistance to CAR T cell therapy. Nat. Rev. Clin. Oncol..

[9] Guedan S., Calderon H., Posey A.D., Maus M.V. (2018). Engineering and design of chimeric antigen receptors. Mol. Ther. Methods Clin. Dev..

[10] Weinkove R., George P., Dasyam N., McLellan A.D. (2019). Selecting costimulatory domains for chimeric antigen receptors: Functional and clinical considerations. Clin. Transl. Immunol..

[11] Picanço-Castro V., Moço P., Mizukami A., Vaz L.D., Pereira M.D.S.F., Silvestre R.N., De Azevedo J.T.C., Bomfim A.D.S., Neto M.S.D.A., Malmegrim K.C.R. (2020). Establishment of a simple and efficient platform for car-t cell generation and expansion: From lentiviral production to in vivo studies. Hematol. Transfus. Cell Ther..

[12] Izsvak Z., Hackett P.B., Cooper L.J., Ivics Z. (2010). Translating Sleeping Beauty transposition into cellular therapies: Victories and challenges. BioEssays.

[13] Hu B., Ren J., Luo Y., Keith B., Young R.M., Scholler J., Zhao Y., June C.H. (2017). Augmentation of antitumor immunity by human and mouse CAR T cells secreting IL-18. Cell Rep..

[14] Spolski R., Leonard W.J. (2014). Interleukin-21: A double-edged sword with therapeutic potential. Nat. Rev. Drug Discov..

[15] Zhang L., Morgan R.A., Beane J., Zheng Z., Dudley M.E., Kassim S.H., Nahvi A.V., Ngo L.T., Sherry R.M., Phan G.Q. (2015). Tumor-infiltrating lymphocytes genetically engineered with an inducible gene encoding interleukin-12 for the immunotherapy of metastatic melanoma. Clin. Cancer Res..

[16] Papadouli I., Mueller-Berghaus J., Beuneu C., Ali S., Hofner B., Petavy F., Tzogani K., Miermont A., Norga K., Kholmanskikh O. (2020). EMA Review of Axicabtagene Ciloleucel (Yescarta) for the treatment of diffuse large B-Cell Lymphoma. Oncology.

[17] Ali S., Kjeken R., Niederlaender C., Markey G., Saunders T.S., Opsata M., Moltu K., Bremnes B., Grønevik E., Muusse M. (2019). The european medicines agency review of Kymriah (Tisagenlecleucel) for the treatment of acute Lymphoblastic Leukemia and diffuse large B-cell Lymphoma. Oncology.

[18] Boissonnas A., Fetler L. (2007). In vivo imaging of cytotoxic T cell infiltration and elimination of a solid tumor. J. Exp. Med..

[19] Craddock J.A., Lu A., Bear A., Pule M., Brenner M.K., Rooney C.M., Foster A.E. (2010). Enhanced tumor trafficking of GD2 Chimeric antigen receptor T cells by expression of the Chemokine receptor CCR2b. J. Immunother..

[20] Harlin H., Meng Y., Peterson A.C., Zha Y., Tretiakova M., Slingluff C., McKee M., Gajewski T.F. (2009). Chemokine expression in melanoma metastases associated with CD8+ T-Cell recruitment. Cancer Res..

[21] Moon E.K., Carpenito C., Sun J., Wang L.-C.S., Kapoor V., Predina J., Powell D.J., Riley J.L., June C.H., Albelda S.M. (2011). Expression of a functional CCR2 receptor enhances tumor localization and tumor eradication by retargeted human T cells expressing a mesothelin-specific chimeric antibody receptor. Clin. Cancer Res..

[22] Idorn M., Straten P. (2018). Chemokine receptors and exercise to tackle the inadequacy of T cell homing to the tumor site. Cells.

[23] Slaney C.Y., Kershaw M., Darcy P.K. (2014). Trafficking of T cells into tumors. Cancer Res..

[24] Griffioen A.W. (2008). Anti-angiogenesis: Making the tumor vulnerable to the immune system. Cancer Immunol. Immunother..

[25] Schaaf M.B., Garg A.D., Agostinis P. (2018). Defining the role of the tumor vasculature in antitumor immunity and immunotherapy. Cell Death Dis..

[26] Zhang J., Endres S., Kobold S. (2019). Enhancing tumor T cell infiltration to enable cancer immunotherapy. Immunotheraphy.

[27] Cho A., Howell V.M., Colvin E.K. (2015). The extracellular matrix in epithelial ovarian cancer—A piece of a puzzle. Front. Oncol..

[28] Kerbel R.S. (2011). Reappraising antiangiogenic therapy for breast cancer. Breast.

[29] Galon J., Costes A., Kirilovsky A., Mlecnik B., Lagorce-Pagès C., Tosolini M., Camus M., Zinzindohoué F., Bruneval P., Cugnenc P.-H. (2006). Type, density, and location of immune cells within human colorectal tumors predict clinical outcome. Science.

[30] Kaushik S., Pickup M.W., Weaver V. (2016). From transformation to metastasis: Deconstructing the extracellular matrix in breast cancer. Cancer Metastasis Rev..

[31] Kim S.T., Jeong H., Woo O.H., Seo J.H., Kim A., Lee E.S., Shin S.W., Kim Y.H., Kim J.S., Park K.H. (2013). Tumor-infiltrating Lymphocytes, tumor characteristics, and recurrence in patients with early breast cancer. Am. J. Clin. Oncol..

[32] Kmiecik J., Poli A., Brons N.H., Waha A., Eide G.E., Enger P. (2013). Øyvind; Zimmer, J.; Chekenya, M. Elevated CD3+ and CD8+ tumor-infiltrating immune cells correlate with prolonged survival in glioblastoma patients despite integrated immunosuppressive mechanisms in the tumor microenvironment and at the systemic level. J. Neuroimmunol..

[33] Li J., Wientjes M.G., Au J.L.-S. (2010). Pancreatic cancer: Pathobiology, treatment options, and drug delivery. AAPS J..

[34] Piersma S.J., Jordanova E.S., Van Poelgeest M.I., Kwappenberg K.M., Van Der Hulst J.M., Drijfhout J.W., Melief C.J., Kenter G., Fleuren G.J., Offringa R. (2007). High number of intraepithelial CD8+ tumor-infiltrating lymphocytes is associated with the absence of lymph node metastases in patients with large early-stage cervical cancer. Cancer Res..

[35] Zhang E., Gu J., Xu H. (2018). Prospects for chimeric antigen receptor-modified T cell therapy for solid tumors. Mol. Cancer.

[36] Martinet L., Le Guellec S., Filleron T., Lamant L., Meyer N., Rochaix P., Garrido I., Girard J.-P. (2012). High endothelial venules (HEVs) in human melanoma lesions. OncoImmunology.

[37] Digre A., Singh K., Åbrink M., Reijmers R.M., Sandler S., Vlodavsky I., Li J.-P. (2017). Overexpression of heparanase enhances T lymphocyte activities and intensifies the inflammatory response in a model of murine rheumatoid arthritis. Sci. Rep..

[38] Theocharis A.D., Skandalis S.S., Gialeli C., Karamanos N. (2016). Extracellular matrix structure. Adv. Drug Deliv. Rev..

[39] Shrimali R.K., Yu Z., Theoret M.R., Chinnasamy D., Restifo N.P., Rosenberg S.A. (2010). Antiangiogenic agents can increase lymphocyte infiltration into tumor and enhance the effectiveness of adoptive immunotherapy of cancer. Cancer Res..

[40] Caruana I., Savoldo B., Hoyos V., Weber G., Liu H., Kim E.S., Ittmann M.M., Marchetti D., Dotti G. (2015). Heparanase promotes tumor infiltration and antitumor activity of CAR-redirected T lymphocytes. Nat. Med..

[41] Burga R.A., Thorn M., Point G.R., Guha P., Nguyen C.T., Licata L.A., DeMatteo R.P., Ayala A., Espat N.J., Junghans R.P. (2015). Liver myeloid-derived suppressor cells expand in response to liver metastases in mice and inhibit the anti-tumor efficacy of anti-CEA CAR-T. Cancer Immunol. Immunother..

[42] Facciabene A., Peng X., Hagemann I.S., Balint K., Barchetti A., Wang L.-P., Gimotty P.A., Gilks C.B., Lal P., Zhang L. (2011). Tumour hypoxia promotes tolerance and angiogenesis via CCL28 and Treg cells. Nature.

[43] Newick K., O’Brien S., Moon E., Albelda S.M. (2017). CAR T cell Therapy for solid tumors. Annu. Rev. Med..

[44] Yao X., Ahmadzadeh M., Lu Y.-C., Liewehr D.J., Dudley M.E., Liu F., Schrump D.S., Steinberg S.M., Rosenberg S.A., Robbins P.F. (2012). Levels of peripheral CD4+FoxP3+ regulatory T cells are negatively associated with clinical response to adoptive immunotherapy of human cancer. Blood.

[45] Gabrilovich D.I., Nagaraj S. (2009). Myeloid-derived suppressor cells as regulators of the immune system. Nat. Rev. Immunol..

[46] Kang C., Jeong S.-Y., Song S.Y., Choi E.K. (2020). The emerging role of myeloid-derived suppressor cells in radiotherapy. Radiat. Oncol. J..

[47] Cassetta L., Pollard J.W. (2020). Tumor-associated macrophages. Curr. Biol..

[48] Kosti P., Maher J., Arnold J.N. (2018). Perspectives on Chimeric antigen receptor T-cell immunotherapy for solid tumors. Front. Immunol..

[49] Anderson A.C., Joller N., Kuchroo V.K. (2016). Lag-3, Tim-3, and TIGIT: Co-inhibitory receptors with specialized functions in immune regulation. Immunity.

[50] Buchbinder E.I., Desai A. (2016). CTLA-4 and PD-1 Pathways: Similarities, differences, and implications of their inhibition. Am. J. Clin. Oncol..

[51] Parry R.V., Chemnitz J.M. (2005). CTLA-4 and PD-1 receptors inhibit T-cell activation by distinct mechanisms. Mol. Cell Biol..

[52] Johnson L.A., June C.H. (2016). Driving gene-engineered T cell immunotherapy of cancer. Cell Res..

[53] Maus M.V., Haas A.R., Beatty G.L., Albelda S.M., Levine B.L., Liu X., Zhao Y., Kalos M., June C.H. (2013). T-cells expressing chimeric antigen receptors can cause anaphylaxis in humans. Cancer Immunol. Res..

[54] Mirzaei H.R., Rodriguez A., Shepphird J., Brown C., Badie B. (2017). Chimeric Antigen Receptors T cell therapy in solid tumor: Challenges and clinical applications. Front. Immunol..

[55] Butte M.J., Pena-Cruz V., Kim M.-J., Freeman G.J., Sharpe A.H. (2008). Interaction of human PD-L1 and B7-1. Mol. Immunol..

[56] Freeman G.J., Long A.J., Iwai Y., Bourque K., Chernova T., Nishimura H., Fitz L.J., Malenkovich N., Okazaki T., Byrne M.C. (2000). Engagement of the Pd-1 immunoinhibitory receptor by a novel B7 family member leads to negative regulation of lymphocyte activation. J. Exp. Med..

[57] Dong Y., Sun Q., Zhang X. (2016). PD-1 and its ligands are important immune checkpoints in cancer. Oncotarget.

[58] Keir M.E., Butte M.J., Freeman G.J., Sharpe A.H. (2008). PD-1 and Its ligands in tolerance and immunity. Annu. Rev. Immunol..

[59] Chambers C.A., Kuhns M.S. (2001). CTLA-4-mediated inhibition in regulation of T cell responses: Mechanisms and manipulation in tumor immunotherapy. Annu. Rev. Immunol..

[60] Egen J.G., Kuhns M.S., Allison J.P. (2002). CTLA-4: New insights into its biological function and use in tumor immunotherapy. Nat. Immunol..

[61] McGowan E., Lin Q., Ma G., Yin H., Chen S., Lin Y. (2020). PD-1 disrupted CAR-T cells in the treatment of solid tumors: Promises and challenges. Biomed. Pharmacother..

[62] Joller N., Kuchroo V.K. (2017). Tim-3, Lag-3, and TIGIT. Curr. Top. Microbiol. Immunol..

[63] Liou G.-Y., Storz P. (2010). Reactive oxygen species in cancer. Free. Radic. Res..

[64] Weinberg F., Ramnath N., Nagrath D. (2019). Reactive oxygen species in the tumor microenvironment: An overview. Cancers.

[65] Yang Z., Guo J., Weng L., Tang W., Jin S., Ma W. (2020). Myeloid-derived suppressor cells—New and exciting players in lung cancer. J. Hematol. Oncol..

[66] Ohl K., Tenbrock K. (2018). Reactive oxygen species as regulators of MDSC-mediated immune suppression. Front. Immunol..

[67] Corzo C.A., Cotter M.J., Cheng P., Cheng F., Kusmartsev S., Sotomayor E., Padhya T., McCaffrey T.V., McCaffrey J.C., Gabrilovich D.I. (2009). Mechanism regulating reactive oxygen species in tumor-induced myeloid-derived suppressor cells. J. Immunol..

[68] Mazzoni A., Bronte V., Visintin A., Spitzer J.H., Apolloni E., Serafini P., Zanovell P., Segal D.M. (2002). Myeloid suppressor lines inhibit T cell responses by an NO-dependent mechanism. J. Immunol..

[69] Pizzino G., Irrera N., Cucinotta M., Pallio G., Mannino F., Arcoraci V., Squadrito F., Altavilla D., Bitto A. (2017). Oxidative Stress: Harms and Benefits for Human Health. Oxidative Med. Cell. Longev..

[70] Klaunig J.E. (2019). Oxidative Stress and Cancer. Curr. Pharm. Des..

[71] Baumann T., Dunkel A., Schmid C., Schmitt S., Hiltensperger M., Lohr K., Laketa V., Donakonda S., Ahting U., Lorenz-Depiereux B. (2020). Regulatory myeloid cells paralyze T cells through cell-cell transfer of the metabolite methylglyoxal. Nat. Immunol..

[72] De La Cruz-López K.G., Castro-Muñoz L.J., Reyes-Hernández D.O., García-Carrancá A., Manzo-Merino J. (2019). Lactate in the regulation of tumor microenvironment and therapeutic approaches. Front. Oncol..

[73] Arab S., Hadjati J. (2019). Adenosine blockage in tumor microenvironment and improvement of cancer immunotherapy. Immune Netw..

[74] Huang Q., Xia J. (2018). MiR-153 suppresses IDO1 expression and enhances CAR T cell immunotherapy. J. Hematol. Oncol..

[75] Rodríguez P.C. (2004). Arginase I production in the tumor microenvironment by mature myeloid cells inhibits T-cell receptor expression and antigen-specific T-cell responses. Cancer Res..

[76] Bronte V., Serafini P., Mazzoni A., Segal D.M., Zanovell P. (2003). L-arginine metabolism in myeloid cells controls T-lymphocyte functions. Trends Immunol..

[77] Cheng J., Zhao L., Zhang Y., Qin Y., Guan Y., Zhang T., Liu C., Zhou J. (2019). Understanding the mechanisms of resistance to CAR T-Cell therapy in malignancies. Front. Oncol..

[78] Han S., Latchoumanin O., Wu G., Zhou G., Hebbard L., George J., Qiao L. (2017). Recent clinical trials utilizing chimeric antigen receptor T cells therapies against solid tumors. Cancer Lett..

[79] Juillerat A., Marechal A., Filhol J.M., Valogne Y., Valton J., Duclert A., Duchateau P., Poirot L. (2017). An oxygen sensitive self-decision making engineered CAR T-cell. Sci. Rep..

[80] Bollong M.J., Lee G., Coukos J.S., Yun H., Zambaldo C., Chang J.W., Chin E.N., Ahmad I., Chatterjee A.K., Lairson L.L. (2018). A metabolite-derived protein modification integrates glycolysis with KEAP1–NRF2 signalling. Nature.

[81] Nokin M.J., Durieux F. (2016). Methylglyoxal, a glycolysis side-product, induces Hsp90 glycation and YAP-mediated tumor growth and metastasis. eLife.

[82] Xu X., Gnanaprakasam J.N.R., Sherman J., Wang R. (2019). A Metabolism toolbox for CAR T therapy. Front. Oncol..

[83] Bollard C.M., Tripic T., Cruz C.R., Dotti G., Gottschalk S., Torrano V., Dakhova O., Carrum G., Ramos C.A., Liu H. (2018). Tumor-specific T-cells engineered to overcome tumor immune evasion induce clinical responses in patients with relapsed hodgkin lymphoma. J. Clin. Oncol..

[84] Chinnasamy D., Yu Z., Kerkar S.P., Zhang L., Morgan R.A., Restifo N.P., Rosenberg S.A. (2012). Local delivery of lnterleukin-12 Using T cells targeting VEGF Receptor-2 Eradicates multiple vascularized tumors in mice. Clin. Cancer Res..

[85] Kloss C.C., Lee J. (2018). Dominant-negative TGF-beta receptor enhances PSMA-targeted human CAR T cell proliferation and augments prostate cancer eradication. Mol. Ther..

[86] Koneru M., Purdon T., Spriggs D., Koneru S., Brentjens R.J. (2015). IL-12 secreting tumor-targeted chimeric antigen receptor T cells eradicate ovarian tumors in vivo. OncoImmunology.

[87] Wallace A., Kapoor V., Sun J., Mrass P., Weninger W., Heitjan D.F., June C., Kaiser L.R., Ling L.E., Albelda S.M. (2008). Transforming growth factor-beta receptor blockade augments the effectiveness of adoptive T-cell therapy of established solid cancers. Clin. Cancer Res..

[88] Law A.M.K., Valdes-Mora F., Gallego-Ortega D. (2020). Myeloid-derived suppressor cells as a therapeutic target for cancer. Cells.

[89] Taylor A., Verhagen J., Blaser K., Akdis M., Akdis C.A. (2006). Mechanisms of immune suppression by interleukin-10 and transforming growth factor-beta: The role of T regulatory cells. Immunology.

[90] Massagué J. (2008). TGFbeta in cancer. Cell.

[91] Yeh H.-W., Lee S.-S., Chang C.-Y., Lang Y.-D., Jou Y.-S. (2019). A new switch for TGFβ in cancer. Cancer Res..

[92] Dahmani A., Delisle J.-S. (2018). TGF-β in T cell biology: Implications for cancer immunotherapy. Cancers.

[93] Dennis K.L., Blatner N.R., Gounari F., Khazaie K. (2013). Current status of interleukin-10 and regulatory T-cells in cancer. Curr. Opin. Oncol..

[94] Hong I.-S. (2016). Stimulatory versus suppressive effects of GM-CSF on tumor progression in multiple cancer types. Exp. Mol. Med..

[95] Bailey K.M., Wojtkowiak J.W., Hashim A.I., Gillies R.J. (2012). Targeting the metabolic microenvironment of tumors. HIV-1 Mol. Biol. Pathog..

[96] Zhang X., Lin Y., Gillies R.J. (2010). Tumor pH and its measurement. J. Nucl. Med..

[97] Zhou Y., Chen X., Cao J., Gao H. (2020). Overcoming the biological barriers in the tumor microenvironment for improving drug delivery and efficacy. J. Mater. Chem. B.

[98] Kato Y., Ozawa S., Miyamoto C., Maehata Y., Suzuki A., Maeda T., Baba Y. (2013). Acidic extracellular microenvironment and cancer. Cancer Cell Int..

[99] Zou W., Chen L. (2008). Inhibitory B7-family molecules in the tumour microenvironment. Nat. Rev. Immunol..

[100] Martinez M., Moon E.K. (2019). CAR T cells for solid tumors: New strategies for finding, infiltrating, and surviving in the tumor microenvironment. Front. Immunol..

[101] Sfanos K.S., Bruno T.C., Meeker A.K., De Marzo A.M., Isaacs W.B., Drake C.G. (2009). Human prostate-infiltrating CD8+T lymphocytes are oligoclonal and PD-1+. Prostate.

[102] Ahmadzadeh M. (2009). Johnson, L.A. Tumor antigen-specific CD8 T cells infiltrating the tumor express high levels of PD-1 and are functionally impaired. Clin. Immunol..

[103] Kusmartsev S., Nefedova Y., Yoder D., I Gabrilovich D. (2004). Antigen-specific inhibition of CD8+ T cell response by immature myeloid cells in cancer is mediated by reactive oxygen species. J. Immunol..

[104] Xiang H., Ramil C.P., Hai J., Zhang C., Wang H., Watkins A.A., Afshar R., Georgiev P., Sze M.A., Song X.S. (2020). Cancer-associated fibroblasts promote immunosuppression by inducing ROS-generating monocytic MDSCs in lung Squamous cell Carcinoma. Cancer Immunol. Res..

[105] Siska P.J., Beckermann K.E., Mason F.M., Andrejeva G., Greenplate A.R., Sendor A.B., Chiang Y.-C.J., Corona A.L., Gemta L.F., Vincent B.G. (2017). Mitochondrial dysregulation and glycolytic insufficiency functionally impair CD8 T cells infiltrating human renal cell carcinoma. JCI Insight.

[106] Wang Y., Jiang H. (2019). An IL-4/21 Inverted Cytokine receptor improving CAR-T cell potency in immunosuppressive solid-tumor microenvironment. Front. Immunol..

[107] Schurich A., Magalhaes I., Mattsson J. (2019). Metabolic regulation of CAR T cell function by the hypoxic microenvironment in solid tumors. Immunotheraphy.

[108] Zhao L., Cao Y.J. (2019). Engineered T Cell Therapy for Cancer in the Clinic. Front. Immunol..

[109] Richman S.A., Nunez-Cruz S., Moghimi B., Li L., Gershenson Z.T., Mourelatos Z., Barrett D.M., Grupp S.A., Milone M.C. (2017). High-affinity GD2-Specific CAR T cells induce fatal encephalitis in a preclinical neuroblastoma model. Cancer Immunol. Res..

[110] Morgan R.A., Yang J.C., Kitano M., E Dudley M., Laurencot C.M., A Rosenberg S. (2010). Case report of a serious adverse event following the administration of T cells transduced with a chimeric antigen receptor recognizing ERBB2. Mol. Ther..

[111] Lamers C.H.J., Sleijfer S., Van Steenbergen S., Van Elzakker P., Van Krimpen B., Groot C., Vulto A., Bakker M.D., Oosterwijk E., Debets R. (2013). Treatment of metastatic renal cell Carcinoma with CAIX CAR-engineered T cells: Clinical evaluation and management of on-target toxicity. Mol. Ther..

[112] Li H., Ding J., Lu M., Liu H., Miao Y., Li L., Wang G., Zheng J., Pei D., Zhang Q. (2020). CAIX-specific CAR-T cells and Sunitinib show synergistic effects against metastatic renal cancer models. J. Immunother..

[113] Ackerman D., Simon M.C. (2014). Hypoxia, lipids, and cancer: Surviving the harsh tumor microenvironment. Trends Cell Biol..

[114] Vaupel P., Mayer A. (2007). Hypoxia in cancer: Significance and impact on clinical outcome. Cancer Metastasis Rev..

[115] Brown J.M., Wilson W.R. (2004). Exploiting tumour hypoxia in cancer treatment. Nat. Rev. Cancer.

[116] Xia A.-L., Wang X.-C., Lu Y.-J., Lu X.-J., Sun B. (2017). Chimeric-antigen receptor T (CAR-T) cell therapy for solid tumors: Challenges and opportunities. Oncotarget.

[117] Van Schalkwyk M.C.I., Papa S., Jeannon J.-P., Urbano T.G., Spicer J.F., Maher J. (2013). Design of a Phase I clinical trial to evaluate intratumoral delivery of ErbB-targeted chimeric antigen receptor T-cells in locally advanced or recurrent head and neck cancer. Hum. Gene Ther. Clin. Dev..

[118] Choi B.D., Suryadevara C.M., Gedeon P., Ii J.E.H., Sanchez-Perez L., Bigner D.D., Sampson J.H., Herndon J.E. (2013). Intracerebral delivery of a third generation EGFRvIII-specific chimeric antigen receptor is efficacious against human glioma. J. Clin. Neurosci..

[119] Sridhar P., Petrocca F. (2017). Regional delivery of chimeric antigen receptor (CAR) T-cells for cancer therapy. Cancers.

[120] Smith T.T., Moffett H.F., Stephan S.B., Opel C.F., Dumigan A., Jiang X., Pillarisetty V.G., Pillai S.P.S., Wittrup K.D., Stephan M.T. (2017). Biopolymers codelivering engineered T cells and STING agonists can eliminate heterogeneous tumors. J. Clin. Investig..

[121] Vignali D., Kallikourdis M. (2017). Improving homing in T cell therapy. Cytokine Growth Factor Rev..

[122] Chow M.T., Luster A.D. (2014). Chemokines in cancer. Cancer Immunol. Res..

[123] Lacy P. (2015). Editorial: Secretion of Cytokines and Chemokines by innate immune cells. Front. Immunol..

[124] McGettrick H.M., Butler L.M., Buckley C.D., Rainger G.E., Nash G.B. (2012). Tissue stroma as a regulator of leukocyte recruitment in inflammation. J. Leukoc. Biol..

[125] Moon E.K., Wang L.-C.S., Bekdache K., Lynn R.C., Lo A., Thorne S.H., Albelda S.M. (2018). Intra-tumoral delivery of CXCL11 via a vaccinia virus, but not by modified T cells, enhances the efficacy of adoptive T cell therapy and vaccines. OncoImmunology.

[126] Kershaw M.H., Wang G., Westwood J.A., Pachynski R.K., Tiffany H.L., Marincola F.M., Wang E., Young H.A., Murphy P.M., Hwu P. (2002). Redirecting migration of T cells to Chemokine secreted from tumors by genetic modification with CXCR2. Hum. Gene Ther..

[127] Di Stasi A., De Angelis B., Rooney C.M., Zhang L., Mahendravada A., Foster A.E., Heslop H.E., Brenner M.K., Dotti G., Savoldo B. (2009). T lymphocytes coexpressing CCR4 and a chimeric antigen receptor targeting CD30 have improved homing and antitumor activity in a Hodgkin tumor model. Blood.

[128] Perera L.P., Zhang M., Nakagawa M., Petrus M.N., Maeda M., Kadin M.E., Waldmann T.A., Perera P.-Y. (2017). Chimeric antigen receptor modified T cells that target chemokine receptor CCR4 as a therapeutic modality for T-cell malignancies. Am. J. Hematol..

[129] Ishitsuka K., Yurimoto S., Kawamura K., Tsuji Y., Iwabuchi M., Takahashi T., Tobinai K. (2017). Safety and efficacy of mogamulizumab in patients with adult T-cell leukemia-lymphoma in Japan: Interim results of postmarketing all-case surveillance. Hematol. Oncol..

[130] Kiesgen S., Chicaybam L., Chintala N.K., Adusumilli P. (2017). Chimeric antigen receptor (CAR) T-cell therapy for Thoracic Malignancies. J. Thorac. Oncol..

[131] Santos A.M., Jung J., Aziz N., Kissil J.L., Puré E. (2009). Targeting fibroblast activation protein inhibits tumor stromagenesis and growth in mice. J. Clin. Investig..

[132] Fu X., Rivera A., Tao L., Zhang X. (2013). Genetically modified T cells targeting neovasculature efficiently destroy tumor blood vessels, shrink established solid tumors and increase nanoparticle delivery. Int. J. Cancer.

[133] Gowrishankar K., Birtwistle L., Micklethwaite K.P. (2018). Manipulating the tumor microenvironment by adoptive cell transfer of CAR T-cells. Mamm. Genome.

[134] Mohammed S., Sukumaran S., Bajgain P., Watanabe N., Heslop H.E., Rooney C.M., Brenner M.K., Fisher W.E., Leen A.M., Vera J.F. (2017). Improving chimeric antigen receptor-modified T cell function by reversing the immunosuppressive tumor microenvironment of pancreatic cancer. Mol. Ther..

[135] Liu X., Ranganathan R., Jiang S., Fang C., Sun J., Kim S., Newick K., Lo A., June C.H., Zhao Y. (2016). A chimeric switch-receptor targeting PD1 augments the efficacy of second-generation CAR T cells in advanced solid tumors. Cancer Res..

[136] Chang Z.L., Lorenzini M.H., Chen X., Tran U., Bangayan N.J., Chen Y.Y. (2018). Rewiring T-cell responses to soluble factors with chimeric antigen receptors. Nat. Methods.

[137] Fedorov V.D., Themeli M., Sadelain M. (2013). PD-1- and CTLA-4-Based inhibitory chimeric antigen receptors (iCARs) divert off-target immunotherapy responses. Sci. Transl. Med..

[138] Wu A., Drake V., Huang H.-S., Chiu S., Zheng L. (2015). Reprogramming the tumor microenvironment: Tumor-induced immunosuppressive factors paralyze T cells. OncoImmunology.

[139] Bollard C.M., Rössig C., Calonge M.J., Huls M.H., Wagner H.-J., Massagué J., Brenner M.K., Heslop H.E., Rooney C.M. (2002). Adapting a transforming growth factor β–related tumor protection strategy to enhance antitumor immunity. Blood.

[140] Foster A.E., Dotti G. (2008). Antitumor activity of EBV-specific T lymphocytes transduced with a dominant negative TGF-beta receptor. J. Immunother..

[141] Löffek S. (2018). Transforming of the tumor microenvironment: Implications for TGF-β inhibition in the context of immune-checkpoint therapy. J. Oncol..

[142] Li S., Siriwon N., Zhang X., Yang S., Jin T., He F., Kim Y.J., Mac J., Lu Z., Wang S. (2017). Enhanced cancer immunotherapy by chimeric antigen receptor–modified T cells engineered to secrete checkpoint inhibitors. Clin. Cancer Res..

[143] John L.B., Devaud C., Duong C.P.M., Yong C.S., Beavis P.A., Haynes N.M., Chow M.T., Smyth M.J., Kershaw M., Darcy P.K. (2013). Anti-PD-1 antibody therapy potently enhances the eradication of established tumors by gene-modified T cells. Clin. Cancer Res..

[144] Ren J., Liu X., Fang C., Jiang S., June C.H., Zhao Y. (2016). Multiplex genome editing to generate universal CAR T cells resistant to PD1 inhibition. Clin. Cancer Res..

[145] Menger L., Sledzinska A., Bergerhoff K., Vargas F.A., Smith J., Poirot L., Pule M., Herrero J., Peggs K.S., Quezada S.A. (2016). TALEN-mediated inactivation of PD-1 in tumor-reactive Lymphocytes promotes Intratumoral T-cell persistence and rejection of established tumors. Cancer Res..

[146] Morgan M., Schambach A. (2018). Engineering CAR-T cells for improved function against solid tumors. Front. Immunol..

[147] Lynn R.C., Weber E.W., Sotillo E., Gennert D., Xu P., Good Z., Anbunathan H., Lattin J., Jones R., Tieu V. (2019). c-Jun overexpression in CAR T cells induces exhaustion resistance. Nature.

[148] Krenciute G., Prinzing B.L. (2017). Transgenic expression of IL15 improves Antiglioma activity of IL13Ralpha2-CAR T cells but results in antigen loss variants. Cancer Immunol. Res..

[149] Adachi K., Kano Y., Nagai T., Okuyama N., Sakoda Y., Tamada K. (2018). IL-7 and CCL19 expression in CAR-T cells improves immune cell infiltration and CAR-T cell survival in the tumor. Nat. Biotechnol..

[150] Zhao Z., Li Y., Liu W., Li X. (2020). Engineered IL-7 receptor enhances the therapeutic effect of AXL-CAR-T cells on triple-negative breast cancer. BioMed Res. Int..

[151] Hoyos V., Savoldo B., Quintarelli C., Mahendravada A., Zhang M., Vera J., Heslop H.E., Rooney C.M., Brenner M.K., Dotti G. (2010). Engineering CD19-specific T lymphocytes with interleukin-15 and a suicide gene to enhance their anti-lymphoma/leukemia effects and safety. Leukemia.

[152] Nishio N., Dotti G. (2015). Oncolytic virus expressing RANTES and IL-15 enhances function of CAR-modified T cells in solid tumors. OncoImmunology.

[153] Alizadeh D., Wong R.A., Yang X., Wang D., Pecoraro J.R., Kuo C.-F., Aguilar B., Qi Y., Ann D.K., Starr R. (2019). IL15 Enhances CAR-T cell antitumor activity by reducing mTORC1 activity and preserving their stem cell memory phenotype. Cancer Immunol. Res..

[154] Cieri N., Camisa B., Cocchiarella F., Forcato M., Oliveira G., Provasi E., Bondanza A., Bordignon C., Peccatori J., Ciceri F. (2013). IL-7 and IL-15 instruct the generation of human memory stem T cells from naive precursors. Blood.

[155] Heczey A., Louis C.U., Savoldo B., Dakhova O., Durett A., Grilley B., Liu H., Wu M.F., Mei Z., Gee A. (2017). CAR T cells administered in combination with Lymphodepletion and PD-1 inhibition to patients with neuroblastoma. Mol. Ther..

[156] Suryadevara C.M., Desai R., Abel M.L., Riccione K.A., Batich K.A., Shen S.H., Chongsathidkiet P., Gedeon P.C., Elsamadicy A.A., Snyder D.J. (2018). Temozolomide lymphodepletion enhances CAR abundance and correlates with antitumor efficacy against established glioblastoma. OncoImmunology.

[157] Bagley S.J., O’Rourke D.M. (2020). Clinical investigation of CAR T cells for solid tumors: Lessons learned and future directions. Pharmacol. Ther..

[158] Hombach A.A., Geumann U., Günther C., Hermann F.G., Abken H. (2020). IL7-IL12 engineered Mesenchymal stem cells (MSCs) improve a CAR T cell attack against colorectal cancer cells. Cells.

[159] Sun S., Hao H., Yang G., Zhang Y., Fu Y. (2018). Immunotherapy with CAR-Modified T cells: Toxicities and overcoming strategies. J. Immunol. Res..

[160] Jackson H.J., Brentjens R.J. (2015). Overcoming antigen escape with CAR T-cell therapy. Cancer Discov..

[161] Maude S.L., Frey N., Shaw P.A., Aplenc R., Barrett D.M., Bunin N.J., Chew A., Gonzalez V.E., Zheng Z., Lacey S.F. (2014). Chimeric antigen receptor T cells for sustained remissions in leukemia. New Engl. J. Med..

[162] Han X., Wang Y., Wei J., Han W. (2019). Multi-antigen-targeted chimeric antigen receptor T cells for cancer therapy. J. Hematol. Oncol..

[163] Ruella M., Barrett D.M., Kenderian S.S., Shestova O., Hofmann T.J., Perazzelli J., Klichinsky M., Aikawa V., Nazimuddin F., Kozlowski M. (2016). Dual CD19 and CD123 targeting prevents antigen-loss relapses after CD19-directed immunotherapies. J. Clin. Investig..

[164] Feng K.-C., Guo Y.-L., Liu Y., Dai H.-R., Wang Y., Lv H.-Y., Huang J.-H., Yang Q.-M., Han W. (2017). Cocktail treatment with EGFR-specific and CD133-specific chimeric antigen receptor-modified T cells in a patient with advanced cholangiocarcinoma. J. Hematol. Oncol..

[165] Hombach A., Rappl G., Abken H. (2019). Blocking CD30 on T Cells by a Dual Specific CAR for CD30 and Colon Cancer Antigens Improves the CAR T cell response against CD30- Tumors. Mol. Ther..

[166] Hegde M., Mukherjee M., Grada Z., Pignata A., Landi D., Navai S., Wakefield A., Fousek K., Bielamowicz K., Chow K.K. (2016). Tandem CAR T cells targeting HER2 and IL13Rα2 mitigate tumor antigen escape. J. Clin. Investig..

[167] Ma L., Dichwalkar T., Chang J.Y., Cossette B., Garafola D., Zhang A.Q., Fichter M., Wang C., Liang S., Silva M. (2019). Enhanced CAR-T cell activity against solid tumors by vaccine boosting through the chimeric receptor. Science.

[168] Becerra C.R., Hoof P., Paulson A.S., Manji G.A., Gardner O., Malankar A., Shaw J., Blass D., Ballard B., Yi X. (2019). Ligand-inducible, prostate stem cell antigen (PSCA)-directed GoCAR-T cells in advanced solid tumors: Preliminary results from a dose escalation. J. Clin. Oncol..

[169] Labanieh L., Majzner R.G., Mackall C.L. (2018). Programming CAR-T cells to kill cancer. Nat. Biomed. Eng..

[170] Roybal K.T., Rupp L.J., Morsut L., Walker W.J., McNally K.A., Park J.S., Lim W.A. (2016). Precision tumor recognition by T cells with combinatorial antigen-sensing circuits. Cell.

[171] Raj D., Yang M.-H., Rodgers D., Hampton E.N., Begum J., Mustafa A., Lorizio D., Garces I., Propper D., Kench J.G. (2018). Switchable CAR-T cells mediate remission in metastatic pancreatic ductal adenocarcinoma. Gut.

[172] Badrinath N., Yoo S.Y. (2019). Recent advances in cancer stem cell-targeted immunotherapy. Cancers.

[173] Ferrandina G., Petrillo M., Bonanno G., Scambia G. (2009). Targeting CD133 antigen in cancer. Expert Opin. Ther. Targets.

[174] Wang Y., Chen M., Wu Z., Tong C., Dai H., Guo Y., Liu Y., Huang J., Lv H., Luo C. (2018). CD133-directed CAR T cells for advanced metastasis malignancies: A phase I trial. OncoImmunology.

[175] Kim S.W., Park H.W., Kim H., Lee S., Choi S.Y., Park Y., Lee S.-W. (2019). Evaluating antitumor activity of Kiatomab by targeting cancer stem cell-specific KIAA1114 antigen in mice. Immune Netw..

[176] Simonetta F., Alvarez M., Negrin R.S. (2017). Natural killer cells in graft-versus-host-disease after Allogeneic hematopoietic cell transplantation. Front. Immunol..

[177] (2019). Natural killer cells for cancer immunotherapy: A new CAR is catching up. EBioMedicine.

[178] Wang W., Wu C.-P., Wu C.-P. (2019). CAR-NK for tumor immunotherapy: Clinical transformation and future prospects. Cancer Lett..

[179] Habib S., Tariq S.M., Tariq M. (2019). Chimeric Antigen Receptor-Natural Killer Cells: The Future of Cancer Immunotherapy. Ochsner J..

[180] CAR NK Cells Clinical Trials 2020. https://clinicaltrials.gov/ct2/results?cond=CAR+NK+cells&term=&cntry=&state=&city=&dist=.

[181] Ren J., Zhang X., Liu X., Fang C., Jiang S., June C.H., Zhao Y. (2017). A versatile system for rapid multiplex genome-edited CAR T cell generation. Oncotarget.

[182] Ruella M., Kenderian S.S. (2017). Next-generation chimeric antigen receptor T-cell therapy: Going off the shelf. BioDrugs.

[183] Qasim W., Zhan H., Samarasinghe S., Adams S., Amrolia P., Stafford S., Butler K., Rivat C., Wright G., Somana K. (2017). Molecular remission of infant B-ALL after infusion of universal TALEN gene-edited CAR T cells. Sci. Transl. Med..

[184] Cao J., Wang G., Cheng H., Wei C., Qi K., Sang W., Zhenyu L., Shi M., Li H., Qiao J. (2018). Potent anti-leukemia activities of humanized CD19-targeted chimeric antigen receptor T (CAR-T) cells in patients with relapsed/refractory acute lymphoblastic leukemia. Am. J. Hematol..

[185] Kim G., Hwang H., Jo Y., Lee B., Lee Y.-H., Kim C.H., Hong C. (2018). Soluble γc receptor attenuates anti-tumor responses of CD8+ T cells in T cell immunotherapy. Int. J. Cancer.

